# Supplementary material for: Benthic diel oxygen variability and stress as potential drivers for animal diversification in the Neoproterozoic-Palaeozoic
Source: Nat Commun. 2025 Mar 21;16:2223. doi: 10.1038/s41467-025-57345-0 (PMC11928486; doi:10.1038/s41467-025-57345-0)
Supplement: Supplementary file 1 — Supplementary Information [file 41467_2025_57345_MOESM1_ESM.pdf]

Supplementary Materials for

Benthic diel oxygen variability and stress as potential drivers for animal diversification in the Neoproterozoic-Palaeozoic

Emma U. Hammarlund[1*], Anuraag Bukkuri[1,2,3], Magnus Norling[4], Mazharul Islam[1], Nicole R. Posth[5], Etienne Baratchart[1], Christopher Carroll[1], Sarah R. Amend[6], Robert A. Gatenby[7], Kenneth J. Pienta[6], Joel S. Brown[7], Shanan Peters[8], Kasper Hancke[4]

[1]Tissue Development and Evolution (TiDE) Group, Department of Experimental Medical Science, Lund University, Lund, Sweden.

[2]Department of Computational and Systems Biology, University of Pittsburgh, Pittsburgh, PA, USA.

[3]Center for Evolutionary Biology and Medicine, University of Pittsburgh, PA, USA.

[4]Norwegian Institute for Water Research (NIVA), Oslo, Norway.

[5]Institute for Geosciences & Natural Resource Management (IGN), Geology Section, University of Copenhagen, Copenhagen, Denmark.

[6]The Cancer Ecology Center, Brady Urological Institute, Johns Hopkins School of Medicine, Baltimore, USA.

[7]Department of Integrated Mathematical Oncology, Moffitt Cancer Center, Tampa, USA.

[8]Department of Geoscience, University of Wisconsin–Madison, Madison, USA.

[*]Corresponding author: emma.hammarlund@med.lu.se

<u>**Contents**</u>

Supplementary Figures

Fig. 1. Paleotemperature comparison.
Fig. 2. Calibration results of biogeochemical model.
Fig. 3. Comparison of modelling results when ppO2 is 4% vs 10%.
Fig. 4. Modelling results for ppO_2 4% (0.2 PAL), varying T & TOC adjustment factor.
Fig. 5. Modelling results for ppO_2 10% (0.5 PAL), varying T & TOC adjustment factor.
Fig. 6. Visualization of a Monte-Carlo simulation.
Fig. 7. *In silico* competition experiments eOSM versus pOSM.
Fig. 8. Extinction times for eOSM and pOSM species.

Supplementary Methods

Biogeochemical Model of diel oxygen dynamics
Model of population dynamics and speed of phenotypic plasticity

Supplementary Tables

Table 1. Model parameters
Table 2. Modelling results when adjusting temperature and TOC adjustment factor, at 4% ppO_2 (20% PAL).
Table 3. Modelling results when adjusting temperature and TOC adjustment factor, at 10% ppO_2 (50% PAL).
Table 4. HIF components in Metazoa, as presented in previous analyses.

Supplementary Discussion

Definitions of Hypoxia
Temperature reconstructions
Molecular dynamics within Hypoxia Inducible Factor (HIF)
Biogeochemical model of diel oxygen dynamics
Model of population dynamics and speed of phenotypic plasticity

References

Supplementary Figures

Supplementary Figure 1: A paleotemperature comparison. Modelled global average surface low-latitude temperature from **a**) clumped isotope data from [1] (1st to 50th quantiles of temperature scenario using shallow marine fossils, planktonic foraminifera, limestone and Neoproterozoic dolomite) and **b**) from modified from [2] (grey field represents ±1 std). Gray field represent ±1 std. Dev. In the upper panel a), he PC/C boundary is marked (thick dashed black line). The records are superimposed with original representations accessible here: Mills et al., 2019: https://doi.org/10.1016/j.gr.2018.12.001.
Bergmann et al., 2022: https://10.1002/essoar.10511918.3.

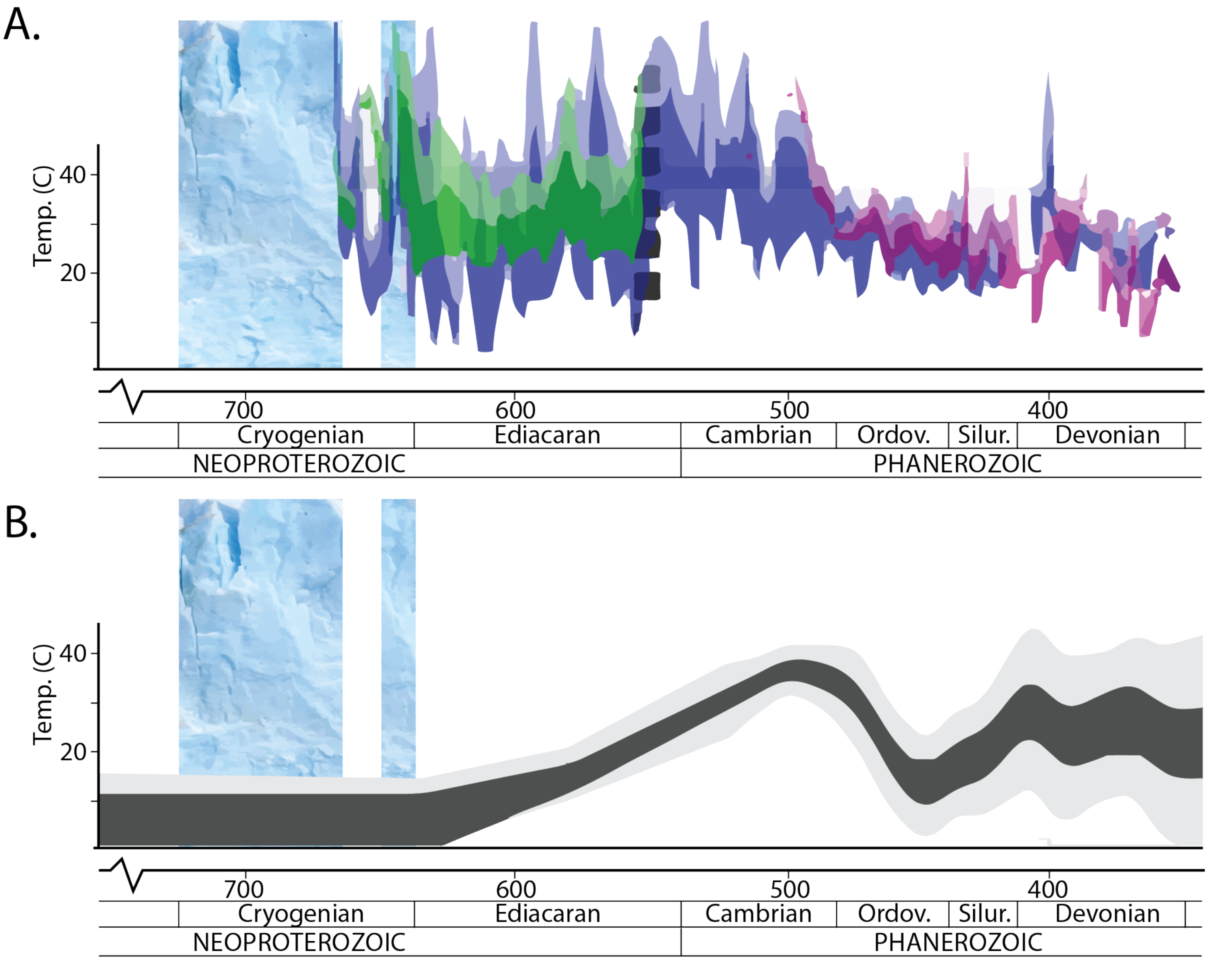

Supplementary Figure 2: A visual representation of the model with annotations and results when the model was calibrated to present-day measured values at a given location (Nivaa Bay, Denmark [3] at 6 different temperatures. Model parameters here: atmospheric oxygen (ppO_2), temperature (T), salinity (S), production Q10 (Q10p), respiration Q10 (Q10r), TOC adjustment factor (c_{oc}), diffusive boundary layer thickness (z_d), and model grid layers.

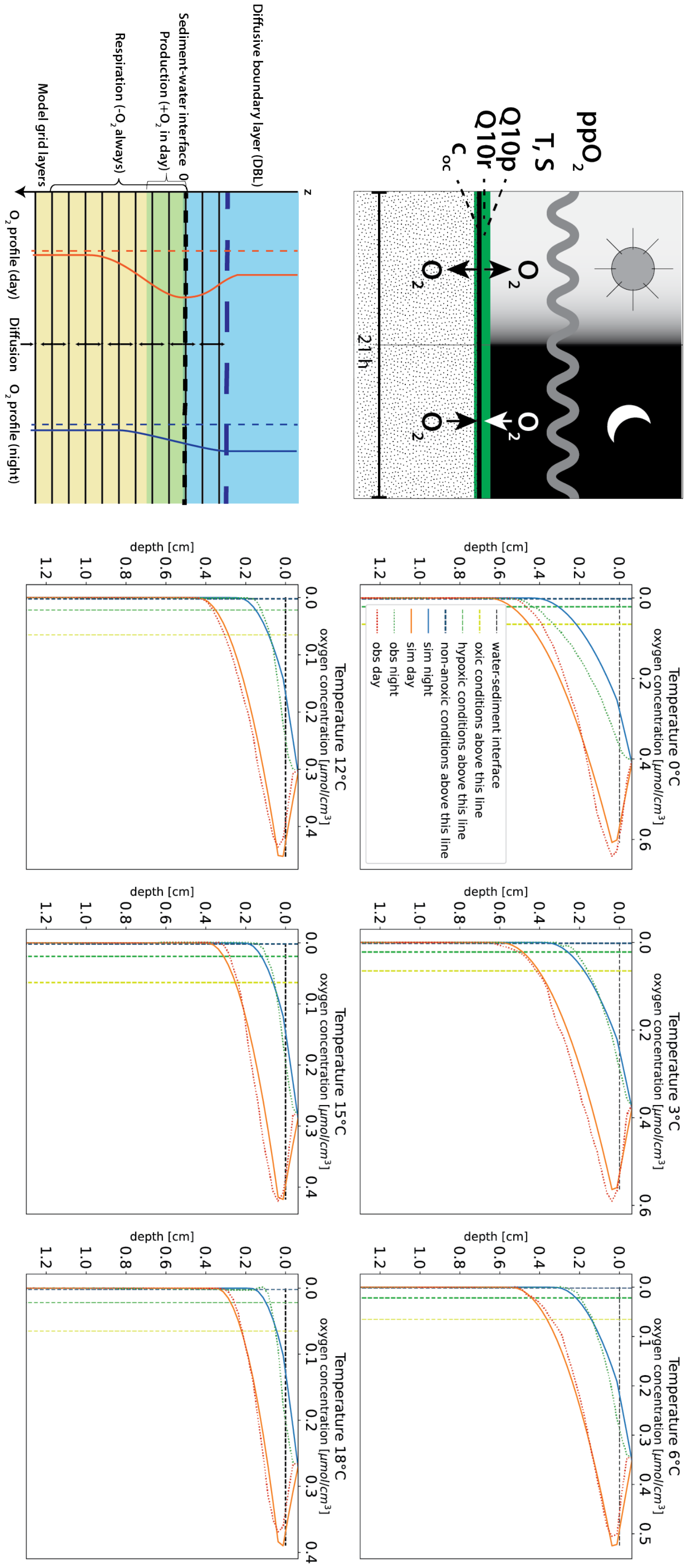

Supplementary Figure 3. Comparison of modelling results when atmospheric oxygen is set (upper panel) at 4% (0.2 PAL) and (lower panel) 10% (0.5 PAL). For other parameters and panel descriptions, see Figure 3 in manuscript. Legend refers to panels A-D.

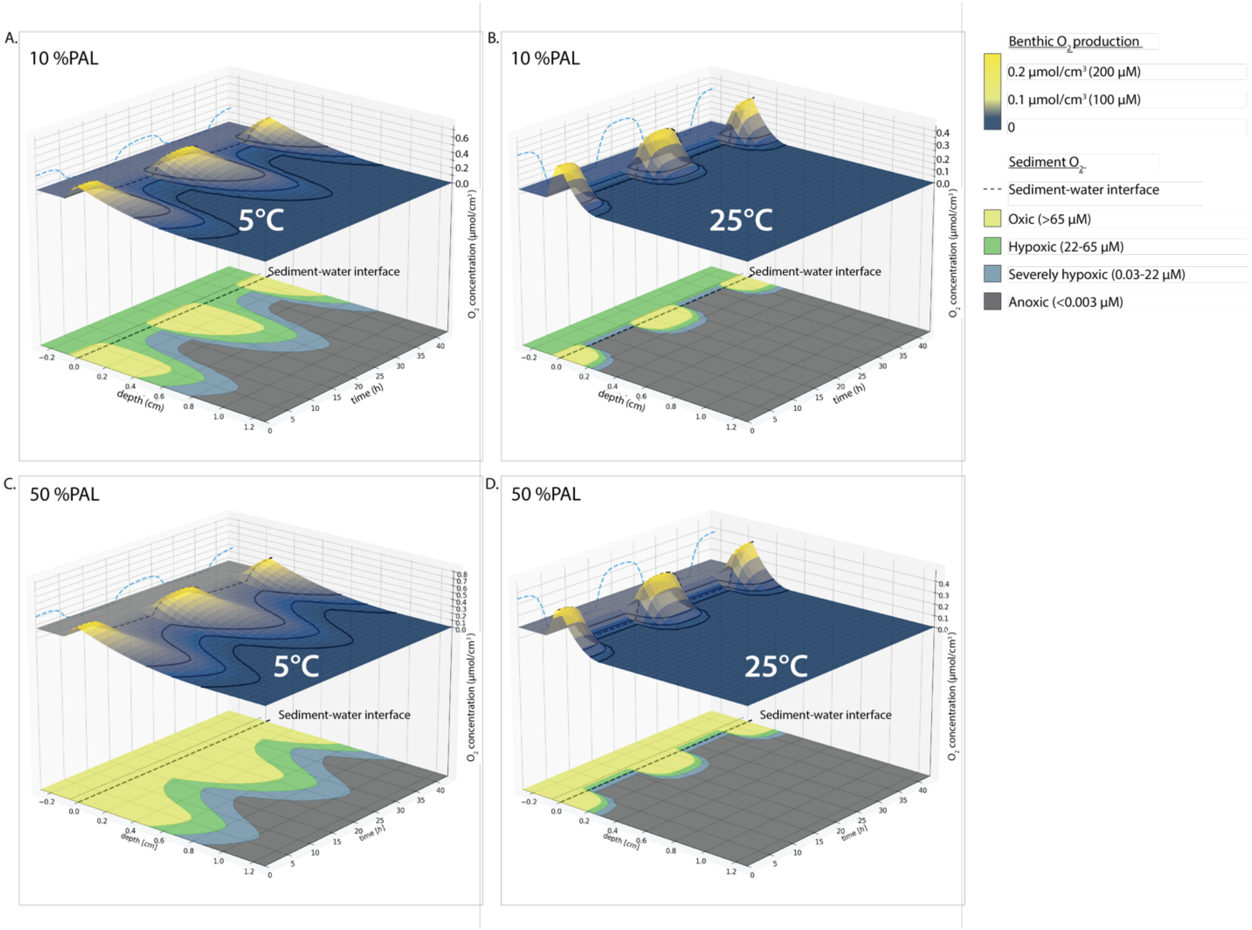

Supplementary Figure 4. Modelling results for ppO2 4% (0.2 PAL) and varying T (5C vs 25C) and TOC adjustment factor (0.1, 0.25, 1). For further modelling, see link to model and files above. Legend refers to all panels.

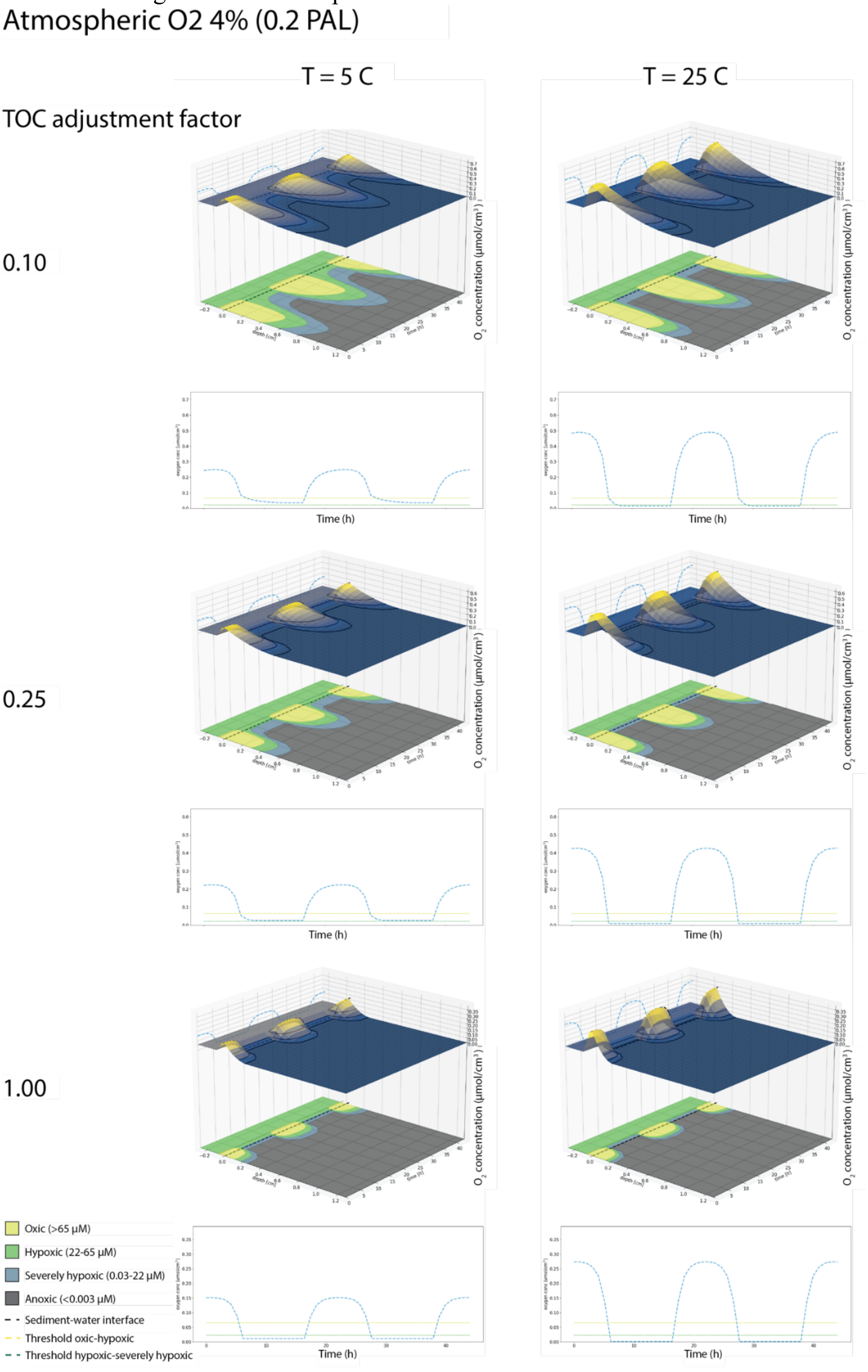

Supplementary Figure 5. Modelling results for ppO2 10% (0.2 PAL) and varying T (5C vs 25C) and TOC adjustment factor (0.1, 0.25, 1). For further modelling, see link to model and files above. Legend refers to all panels.

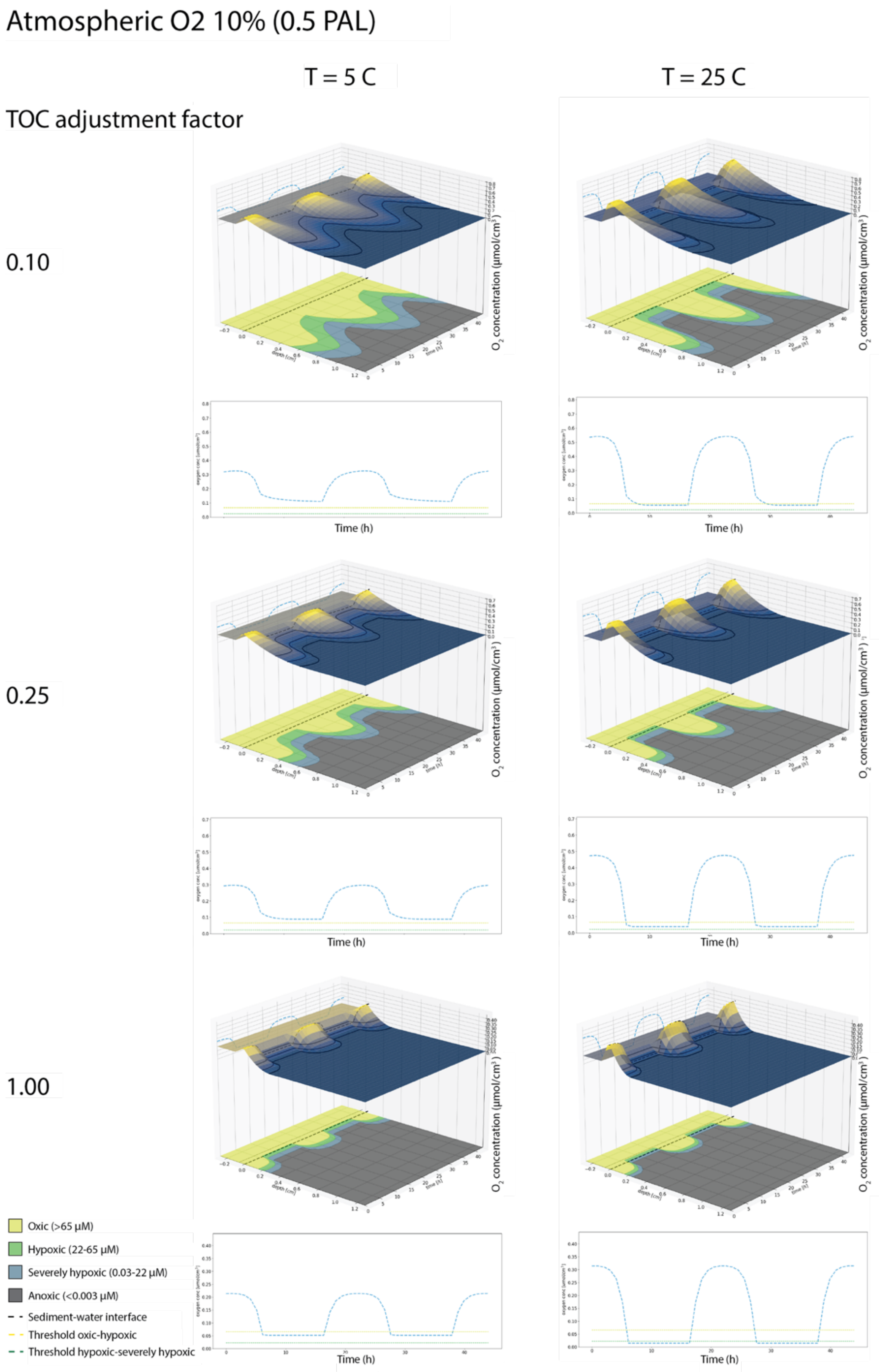

Supplementary Figure 6. Visualization of a Monte-Carlo simulation (50 000 times) testing ranges within the parameters *pp*O_2 (atmospheric O_2 partial pressure, 2-10), prodQ_{10} (O_2 production response to a 10°C change in temperature, 1.5-2.5), respQ_{10} (respiration response to temperature, 1.5-2.5), toc1 (TOC adjustment factor in a cold scenario, 0.1-1) and temperature2 (temperature in hot scenario, 25 °C to 35 °C). The visualization demonstrates when two criteria are fulfilled (grey-black fields):

1) Night concentration in the cold scenario is at least severely hypoxic (or more oxygenated).

2) Days in hot scenario are oxic and nights anoxic, and the transition time between the two is less than 1 h.

The plots below the diagonal are the 'success density' function plotted over the parameter space, projected down to the subspace of two parameters at a time, *i.e.,* the contour plot is darker when the two criteria are more often fulfilled. The plots on the diagonal axis show the same success density projected down to the space of one parameter at a time, shown as a histogram.

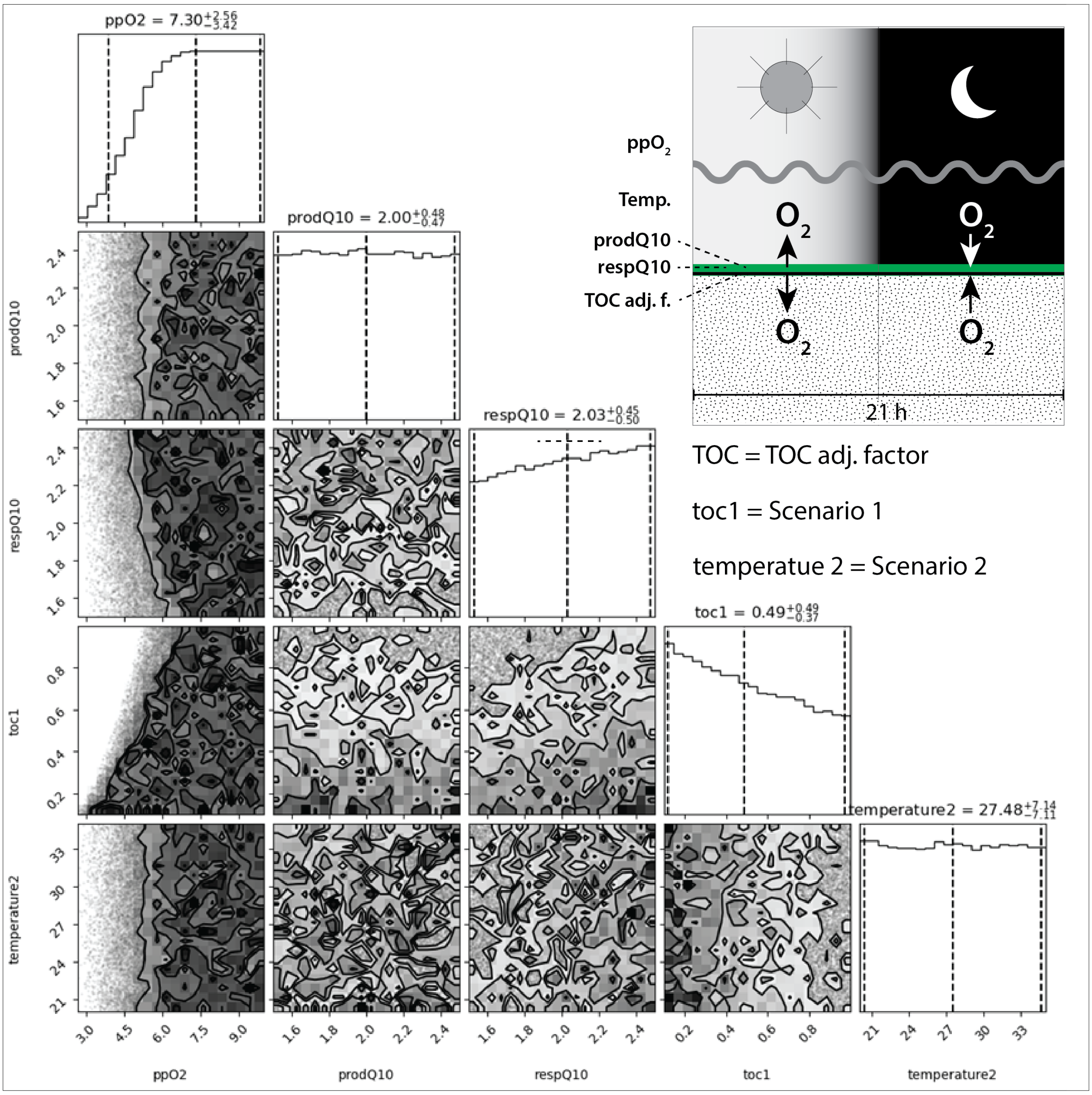

Supplementary Figure 7. *In Silico* Competition Experiments between eOSM and pOSM species for a range of sensing capabilities. Using our *G* function model of Lotka-Volterra competition as described in the main text, we ran a series of simulations competing pOSM and eOSM species for a range of sensing abilities for the pOSM species (s_1) and eOSM species (s_2). To ensure that the eOSM species always had a more efficient oxygen sensing mechanism than the pOSM species, we enforced the constraint: $s_2 > s_1$. The results of these competition experiments under the cases of stochastic, periodic, and stochastic+periodic fluctuations are depicted below. Using our *G* function model of Lotka-Volterra competition as described in the main text, we ran a series of simulations competing pOSM and eOSM species for a range of sensing abilities for the pOSM species (s_1) and eOSM species (s_2). To ensure that the eOSM species always had a more efficient oxygen sensing mechanism than the pOSM species, we enforced the constraint: $s_2 > s_1$. The results of these competition experiments under the cases of stochastic, periodic, and stochastic+periodic fluctuations are depicted below. Each entry in the lower triangular part of the matrices represents the results of a competition experiment. Squares shaded in white represent coexistence, those in red represent extinction of the pOSM species, and those in blue capture extinction of the eOSM species. The darker the shade, the longer the time to extinction.

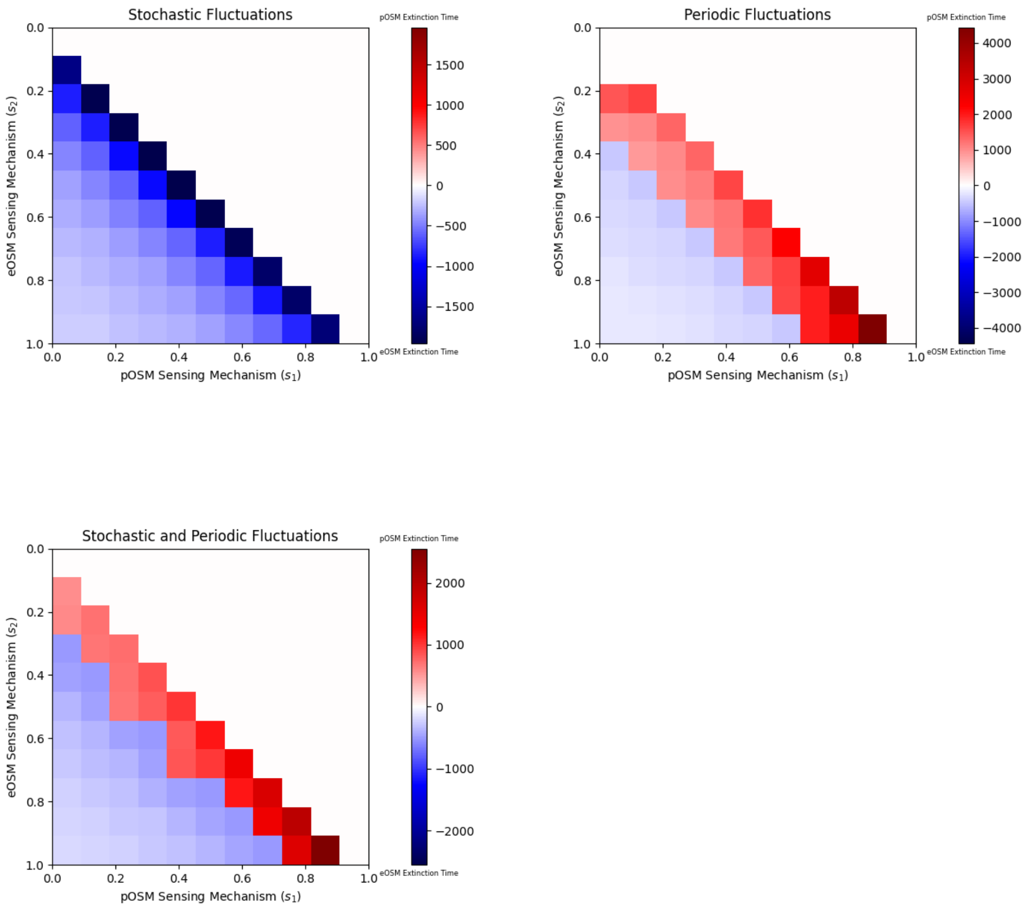

Supplementary Figure 8. Extinction times for *eOSM* or *pOSM* species. A total of 33 simulations across three environmental conditions with eleven different costs of the oxygen sensing mechanism show which species wins out across this range. For each of these simulations, the competition experiment is run until one of the species goes extinct while the other remains extant. Circles, squares, and triangles represent simulation results across environments characterized by stochastic, periodic, and stochastic and periodic oxygen fluctuations, respectively. Blue (red) coloured shapes represent *eOSM* (*pOSM*) extinction events and denote at what time the corresponding population went extinct (*i.e.* the other remains extant). The results show that the t *eOSM* can bear a certain cost for the efficient oxygen sensing in environments with periodic fluctuations and in environments with stochastic and period fluctuations. If the environment offers stochastic fluctuations, *eOSM* can only carry a small cost for efficient oxygen sensing (<0.01) that, if balanced by a benefit, allows them to persist for longer than *pOSM*.

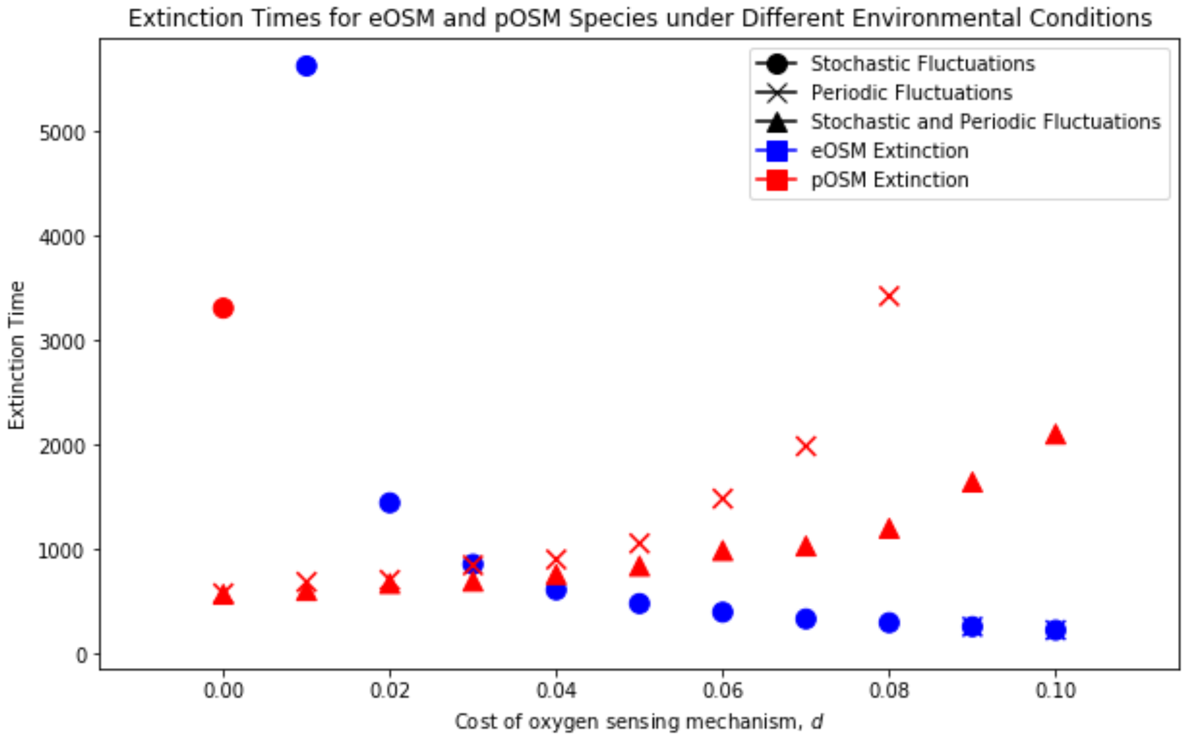

Supplementary Methods
Biogeochemical Model of diel oxygen dynamics

The model solves the 1D diffusion partial differential equation, Eq 1:
(1)

$$\frac{\partial C(z,t)}{\partial t} = \frac{\partial}{\partial z}\left(D(z)\frac{\partial C(z,t)}{\partial z}\right) + p(z,t) - r(C,z,t)$$

Where $C(z,t)$ is the oxygen concentration at depth z and time t, $D(z)$ is the diffusion coefficient, $p(z,t)$ is the oxygen production term, and $r(C,z,t)$ is the oxygen consumption (respiration) term. The equation is in essence a basic partial differential equation that follows Fick's first and second law for diffusive transport (more details are given in Middelburg 2019, Box 1.1, p. 6)[4].

The diffusion coefficient is computed as, Eq 2:
(2)

$$D(z) = \begin{cases} D_0, & z \geq 0 \\ D_0\theta, & z < 0 \end{cases}$$

Where θ is the sediment porosity and $D_0 = D_0(T,S)$ depends on temperature T and salinity S, computed as in [5], and using computation of dynamic viscosity [6]. Temperature and salinity are held constant throughout each single model run but can be different in different scenarios.

Respiration is computed using the following formula, Eq 3:
(3)

$$r(C,z,t) = \begin{cases} 0, & z \geq 0 \\ R, & z < 0, C \geq C_r \\ R\dfrac{C}{C_r}, & z < 0, C < C_r \end{cases}$$

I.e., inside the sediment r is constant unless C falls below the (small) threshold C_r. Temperature-imposed activity changes are often quantified by the so-called 'Q_{10} factor'. The Q_{10} describes the relative rate of increase for a temperature increase of 10°C and is calculated using a formula based on, for example, the apparent activation energy. Since a respiration or production rate has a temperature-dependent response, the rate R is adjusted for temperature using a Q_{10} formula [3,7,8], Eq 4:
(4)

$$R = R_0 c_{oc} Q_{10,r}^{\frac{T-20}{10}}$$

The parameter c_{oc} is an adjustment factor for differences in concentration of organic carbon or biomass between different scenarios. It is set to 1 as a default.

Production is set to be, Eq 5:
(5)

$$p(z,t) = \begin{cases} Q_{10,p}^{\frac{T-20}{10}} M(1 - e^{-\frac{mL}{M}}) \frac{1}{z_p}, & z < 0 \text{ and } z \geq z_p \\ 0, & \text{otherwise} \end{cases}$$

Here M is a maximal production rate (at reference temperature), m is maximal light utilization, and $L = L(t)$ is time-dependent luminosity. Production only happens in the top layer of thickness z_p . Luminosity is computed from global radiation and modified to only accommodate the Photosynthetically Active Radiation (PAR). Global radiation is estimated based on standard formulas[9], but is modified to accommodate for a 21-hour day when run for prehistorical scenarios[10]. Since we are only interested in the general dynamics, cloud cover and attenuation in sea water is set to be constant.

The boundary conditions are set so that $C(-z_d, t)$ is constant for all t, equal to full saturation, depending on atmospheric partial pressure of O_2, salinity and temperature, computed as in [11]. Here z_d is the thickness of the diffusive boundary layer. Moreover $C(z_D, t) = 0$, where z_D is the maximal depth of the modeled domain.

The partial differential equation is solved using a standard finite difference scheme [12] with constant z-resolution resulting in a scheme, Eq 6:

(6)

$$\frac{d\boldsymbol{C}}{dt} = A\boldsymbol{C} + \boldsymbol{f}(\boldsymbol{C}, t)$$

Where $\boldsymbol{C} = \{C_i\}$ is a vector of concentrations at each grid point in the z-direction, A is a tridiagonal matrix coming from the discretization of the differential operators, and $\boldsymbol{f}$ is a time dependent vector of sources and sinks accommodating the respiration and production. This ODE system is solved using an adaptive Runge-Kutta 4 scheme (DASCRU)[13].

Sources

The model is implemented using the Mobius framework [14], and the source code can be found here: https://github.com/NIVANorge/Mobius/tree/master/Modules/SedimentOxygen. Files used for running the model can be found here:
https://github.com/NIVANorge/Mobius/tree/master/Applications/SedimentOxygen

Scenario parameters, see **Table S1**.

Monte-Carlo simulation

Visualization of a Monte-Carlo simulation (50 000 times) testing ranges within the parameters ppO2 (2-10), prodQ10 (1.5-2.5), respQ10 (1.5-2.5), TOC adjustment factor in cold 'Cryogenian' scenario (0.1-1) and temperature in hot 'Cambrian' scenario (20C-35C), see **Supplementary Fig. 6**). The parameters were drawn from a uniform distribution using Latin Hypercubes. The visualization demonstrates when the two criteria 1) Night concentration in the cold scenario is at least severely hypoxic, and 2) Days in hot scenario are oxic and nights anoxic, and the transition between the two is less than 1 h, are fulfilled. The plots are projections of the density of successful outcomes within the given parameter space. The histograms along the diagonal show the density of successful outcomes when projected down to one of the parameter axes at a time, while the grey-black squares are projections of the density down to two parameter axes at a time (black means 100% successful outcomes).

The source code can be found at here and files used for running the model can be found at here.

Model of population dynamics and speed of phenotypic plasticity

We use a mathematical modelling framework called *G functions* that is grounded in evolutionary game theory [15]. *G functions* attempt to simultaneously capture the ecological (population) and, in our case, the phenotype (strategy) dynamics of species using a system of ordinary differential equations. In other words, the framework allows us to track both how the population sizes of the species change over time as well as how their strategies evolve in response to changing environments. The population dynamics for each species is given by Eq 7:

(7)

$$\frac{dx_i}{dt} = x_i G(\mathrm{v}, \mathbf{u}, \mathbf{x})|_{v=u_i}$$

where the fitness generating function, $G(v, \boldsymbol{u}, \boldsymbol{x})$, describes the per capita growth rate of an individual as a function of its own phenotype (strategy), *v*, the phenotypes of the species in the population, $\mathbf{u}$, and the population sizes of each species, **x**.

In this case, the relevant phenotype describes a continuum between anaerobic and aerobic metabolisms within the cells of the multicellular organism. Thus, this equation captures the change in the population size of a given species as a product of its current population size and its per capita growth rate. The ability of the organism to temporally match its cellular metabolism to the particular conditions of dissolved oxygen would lead to higher fitness than having large temporal mismatches. For example, both animals and plants demonstrate functionally convergent OSMs that allow spatiotemporal recognition and response to fluctuations in oxygen [16 and refs within]. We assume that the organisms exhibit phenotypic plasticity and can modify their strategy over time. We assume that this change is dependent on (1) the slope of the selection gradient and (2) how fast the species can change in response to the gradient. The slope of the selection gradient is given by $\frac{dG}{dv}$: the further a species is from its optimal strategy in an environment, the steeper this slope will be. How fast an individual can scale the gradient depends on its oxygen sensing mechanism: the more developed this is, the more quickly a species can respond to the selection gradient. Putting these components together, we derive the equation for an individual's strategy dynamics as, Eq 8:

(8)

$$\frac{du_i}{dt} = s_i \frac{dG}{dv}|_{v=u_i}$$

where s_i is a measure of oxygen sensing. Before running simulations, we must define our fitness generating function, *G*. As our modelling base, we use Lotka-Volterra competition equations. Our equations assume that competition among species is independent of their strategies. In other words, all individuals have the same adverse impacts on each other and compete equally in a density-dependent fashion. This leads to growth in a logistic manner, wherein the carrying capacity depends on how well adapted the species is to its (continually changing) environment. In addition, we assume a linear cost of oxygen sensing--the more efficient an oxygen sensing system is, the more costly it is for an organism to maintain it. The

eOSM and pOSM species are differentiated solely by the eOSM species having a higher capacity for oxygen sensing, *s,* than the pOSM species. Thus, the eOSM species can switch its cellular metabolism more rapidly than the pOSM species, allowing it to more effectively match its environment, but paying an energetic cost for maintaining the system that allows it to do so. The Lotka-Volterra competition equations we use are the following, Eq 9:
(9)

$$G(v,u,x) = \frac{r}{K(v)}\left[K(v) - \sum_{j=1}^{n} x_j\right] - ds$$

There are a couple things to notice here. First, note that carrying capacity is a function of the focal individual's strategy. Namely, as described in the below equation, the closer the individual is to the "optimal strategy", γ, the higher the carrying capacity is, Eq 10:
(10)

$$K(v) = K_m exp\left[-\frac{(v-\gamma)^2}{2\sigma_k^2}\right]$$

Deviations from γ decrease the carrying capacity in a Gaussian fashion. One assumption we make is that competition among species is independent of strategy; in other words, all species have the same adverse impacts on each other. Finally, we include a cost of oxygen sensing through the last term in the *G* function.

We simulate the ecological response when the daily, local, and sedimentary oxygen fluctuations changed successively between a scenario without and then with the benthic sandy shallow niche. There are two main components to oxygen fluctuations: a periodic, cycling oxygen fluctuation due to the production and respiration of benthic microalgae and a stochastic fluctuation due to nutrient cycling. Although a successive increase in global temperature, we here visualize the event as a switch.

This global warming dramatically increased the amplitude of periodic fluctuations. Additionally, the warmer climate would also have enhanced carbon recycling in the water column, through nutrient cycling, algal blooms, and carbon remineralization. To investigate these overall effects of the increased amplitude of oxygen fluctuations in the sediment, we perform three simulations, in which we let γ vary in a sinusoidal fashion with added stochastic noise. We simulate this for 500-time steps to capture dynamics before the early Palaeozoic flooding. Then, for the Cambrian Period, we increase the periodic fluctuation, stochastic fluctuations, and both. In the first simulation, we increase the amplitude of periodic fluctuations while keeping stochasticity constant. In the second simulation, we increase stochastic fluctuations while keeping the amplitude of periodic fluctuations constant. In the last simulation, we increase both the amplitude of periodic fluctuations and the stochasticity.

The parameter values are as follows:
r, growth rate: 0.25
d, death rate: 0.04
s_1, evolvability of *pOSM*: 0.2
s_2, evolvability of *eOSM*: 0.5
K_m, maximal carrying capacity: 100

σ_k^2, breadth of carrying capacity: 12.5

Source code can be found in a separate file.

The simulation was run with different costs and show which species wins out across this range. It demonstrates a cross-over point around the (theoretical) cost of d=0.04. This shows that as the cost of the oxygen sensing mechanism increases, the benefit of efficient oxygen sensing mechanisms goes down in all environments (**Supplementary Fig. 8**).

Supplementary Tables

Supplementary Table 1. Parameter values used in the scenarios. The model validation column are parameter values calibrated to measured concentrations in Nivaa Bay, Denmark [3]. Parameter ranges are tested in the Monte Carlo simulation for both scenarios, except the temperature range (tested for historic scenario 2; **) and TOC adjustment factor (tested for scenario 1; *).

Name	Symbol	Unit	Model validation (Denmark)	Range today	Ref	Hist. scen. 1	Hist. scen. 2	Range tested in Monte Carlo simulation
Thickness of diffusive boundary layer	z_d	cm	0.05	0.02-0.07	[17]	0.05	0.05	
Atmospheric partial pressure		% atm	21	na		4	4	2% - 10%
Salinity	S	PSS-78	17	0-35		35	35	
Temperature	T	°C	0-18	0-50		5	25	20°C- 35°C (**)
Day length		hours	24	na		21	21	
Maximum light utilization	m	μmol[O_2]/μmol[photons]	10^{-9}	$2.8*10^{-10}$ – $5.6*10^{-9}$	[18,19]	10^{-9}	10^{-9}	
Maximum production	M	μmol[O_2]/(cm^2 s)	$6·10^{-5}$			$6·10^{-5}$	$6·10^{-5}$	
Respiration	R_0	μmol[O_2]/(cm^3 s)	$1.1·10^{-4}$	$2*10^{-5}$ – $37*10^{-5}$	[20,21]	$1.1·10^{-4}$	$1.1·10^{-4}$	
TOC		wt%	4	0.5-5.0	[22,23]	1	4	
TOC adjustment factor	c_{oc}		1			0.25	1	0.1-1 (*)
Production Q_10	$Q_{10,p}$		1.4	1.5-3.5	[18,24]	2	2	1.5-2.5
Respiration Q_10	$Q_{10,r}$		2.6	1.5-2.5	[19,25]	2	2	1.5-2.5

Supplementary Table 2. Modelling results when adjusting temperature and TOC adjustment factor, at 4% ppO2 (20% PAL).

Temp (C)	TOC	Daytime max O2 (mM)	Oxic-hypoxic trans. time (h)	Nighttime min O2 (mM)	Severely hypoxic to anoxic interval (h)	SI Fig
5	0.10	0.24	10.56	0.04	0.00	4
	0.25	0.22	11.74	0.02	0.00	
	1.00	0.15	0.68	0.01	11.06	
25	0.10	0.47	1.92	0.02	9.04	4
	0.25	0.42	0.34	0.01	11.23	
	1.00	0.27	0.26	0.00	11.32	

Supplementary Table 3. Modelling results when adjusting temperature and TOC adjustment factor. at 10% ppO2 (50% PAL).

Temp (C)	TOC	Daytime max O2 (mM)	Oxic-hypoxic trans. time (h)	Nighttime min O2 (mM)	Severely hypoxic to anoxic interval (h)	SI Fig
5	0,10	0,33		0,13		5
	1,10	0,30		0,09		
	2,10	0,22	11,40	0,05		
25	3,10	0,53	9,46	0,06		5
	4,10	0,49	11,32	0,05		
	5,10	0,32	0,26	0,02	11,07	

Supplementary Table 4. HIF components in Metazoa, as presented in previous analyses. Protein sequences are collected from NCBI Refseq, JGI, Uniprot, reference 37 and MGP portal (*M. leidyi*).

Species	P564	P402	NTAD	CTAD	FIH site	Phyl.	Ref 37	Ref 38	New ana.	Protein seq
A. queenslandica	N	N	N	N	N	Porif.	✓		✓	NCBI refseq XP_011403284
T. Adherence	Y*	N	N	N	N	Placoz.	✓	✓	✓	Triad_62354
M. leidyi	N	N	N	N	N	Ctenop.	✓	✓	✓	Mle_03195a
N. vectensis	N	N	N	Y	N	Cnid.	✓		✓	Nemve1_160110
L. gigantea	N	Y	N	Y	N	Bilat., Loph., moll.	✓		✓	Lotgi1_159865
S. maritima	N	N	N	Y	N	Bilat., Ecd., Arth., Myriap.	✓		✓	T1JJ64_STRMM
D. pulex	N	N	N	N	N	Bilat., Ecd., Arth., Crust.	✓		✓	Dappu1_309730
D. melanogaster	Y	N	N	N	N	Bilat., Ecd., Nem.	✓	✓	✓	SIMA_DROME
A. gambae	N	N	Y	N	N	Bilat., Ecd., Arth., Insec.	✓		✓	A0NDQ9_ANOGA
B. mori	N	Y	N	N	N	Bilat., Ecd., Arth., Insec.	✓		✓	uniprot A0A8R2R451
T. castaneum	Y	Y	N	Y	Y	Bilat., Ecd., Arth., Insec.	✓		✓	D6WMG7_TRICA
D. ponderosae	Y	Y	N	N	Y	Bilat., Ecd., Arth., Insec.	✓		✓	NCBI refseq XP_048523748.1
N. vitripennis	Y	Y	N	N	N	Bilat., Ecd., Arth., Insec.	✓		✓	NCBI refseq XP_016840589.1
A. pisum	N	Y	N	Y	Y	Bilat., Ecd., Arth., Insec.	✓		✓	NCBI refseq XP_008186097.1
C. elegans	Y	N	N	N	N	Bilat., Ecd., Arth., Insec.	✓		✓	CAEEL_ G5EGD2
S. purpuratus	Y	Y	Y	Y	Y	Chord., Ech., Amb.	✓		✓	NCBI refseq XP_030854578.1
B. floridae	Y	Y	N	N	Y	Chord., Ceph. Chord.	✓		✓	A0A9J7MRS7_BRAFL
C. intestinalis	N	Y	N	Y	Y	Chord., Ceph. Chord.	✓		✓	Cin_004277

*Differs with new analysis

Ref 37: Graham and Presnell 2017

Ref 38: Mills et al., 2018

Supplementary Discussion

Definitions of Hypoxia

The term hypoxia was originally used to describe internal stress on an animal [26]. However, hypoxia has come to describe the external ocean medium which we also do here [27-29]. The specific definitions of anoxic to oxic conditions used here are: anoxic <0.02 μM, severely hypoxic between 0.02 and 22 μM, hypoxic between 22 and 65 μM, and oxic >65 μM[28].

Temperature reconstructions

Several efforts with modeling and oxygen isotope palaeothermometry attempt to reconstruct how temperatures shifted between the Cryogenian and Paleozoic[1,30-32] (**Supplementary Figure 1**). Although their resolution and pace of temperature fluctuations in this time span vary, we chose to visualize the general temperature shift with data from Mills et al., 2019 (**Figure 1**, also **Supplementary Figure 1B**). It is worth noting that even if the clumped isotope data from Bergmann, et al. [1] predict spikes of warmer temperatures in the post-glacial Marinoan, the reconstruction also presents a significant increase at the PC/C boundary (**Supplementary Figure 1A**). Also, temperatures reconstructed from clumped isotopes tend towards the high end (approaching an unreasonable 60°C in the spikes), indicating that the proxy likely needs further calibration. If the reconstructed temperatures are exaggerated, the post-glacial Marinoan temperatures are in the greenhouse zone throughout. Regardless, we chose to present the data from Mills et al., 2019 to simply emphasize that temperatures in general increase over the PC/C boundary.

Molecular dynamics within Hypoxia Inducible Factor (HIF)

HIF (Hypoxia Inducible Factors) are a group of bHLH-PAS (basic Helix Loop Helix – Per/Arnt/SIM) proteins that are constitutively expressed in mammalian cells and act mainly as transcription factors and are key regulators of hypoxia. On protein level, different HIFs share over 50% amino acid homology[33] and can be rapidly degraded under normoxic condition with a half-life of less than 5 minutes[34]. Only under certain hypoxic condition are they stabilized through two proline hydroxylation sites which flanks a special region called oxygen dependent degradation domain (ODDD), which when removed stabilizes HIF even in the presence of oxygen[35]. Otherwise under normoxia, above-mentioned proline residues are hydroxylated by Prolyl hydroxylase enzymes (PHDs, which are directly oxygen dependent) and recognized by Von-Hippel-Lindau protein (pVHL) that marks the protein for ubiquitin mediated proteasomal degradation (by complex formation with other partners)[36,37]. Under Hypoxia, HIFs are translocated to nucleus and binds on specific regions on DNA to activate various responsive genes through their transactivation domains (NTAD and CTAD). Further control is also embedded within CTAD, that has specific site for binding cofactors that are required for transcription of target genes.[38] Previous studies discussed these various features in similar animals from different perspectives[39,40]. Here, a near full scope of the variation within HIF/HIF-like molecule is generated which are presented in **Table S4**. HIF-like molecules present in different animals are compared by multiple sequence alignment (MSA) against human HIF1A and variable components are pointed out in different columns, which also worked as a basis for panel 4F. Proteins were not drawn in scale and the position of the components are approximate. A maximum diversity of components to the OSM is depicted based on detailed mapping of CTAD/NTAD (except the one from *Bombyx mori*) from [39], of P402/P564 from [40], and from the MSA (in addition to CTAD/NTAD for *Bombyx mori*).

Biogeochemical Model of diel oxygen dynamics

The model was calibrated to present-day measured values at a given location (Nivaa Bay, Denmark [3] at 6 different temperatures (**Supplementary Fig. 2**).

The model was run with temperatures set for 5° C and 25° C, where each is tested for three different loads of TOC (TOC adjustment factor from low=0.1, moderate=0.25, high=1), see **Supplementary Fig. 4** (for ppO2 at 4% or 20% PAL) **and Supplementary Fig. 5** (for ppO2 set at 10% or 50% PAL). From these different scenarios, results are presented in terms of Daytime maximum O_2 (mM), Hypoxia transition time (h), Nighttime minimum O_2 (mM), and the extent of the Severely Hypoxic-Anoxic interval (h), see **Supplementary Table 2** (for ppO2 at 4% or 20% PAL) and **Supplementary Table 3** (for ppO2 set at 10% or 50% PAL).

Model of population dynamics and speed of phenotypic plasticity

We performed a sensitivity test of the G-function model. The trends observed in the plots of eco-evolutionary dynamics in presented in the manuscript hold generally for a wide range of parameter values (**Supplementary Fig. 7**). Under the periodic fluctuation and periodic and stochastic fluctuation cases, when sensing capabilities are similar between the eOSM and pOSM species, the pOSM organisms are always driven to extinction. However, when the difference in sensing capabilities is large, the eOSM species goes extinct before or immediately after the Cambrian—the benefits of evolutionarily tracking severe environmental fluctuations is not realized. This is due to the trade-off between the cost of a sensing mechanism and the benefits it provides. If the cost of an efficient sensing mechanism is too high, or alternatively the oxygen fluctuations too modest, the pOSM is favoured. Conversely, low costs and large fluctuations favour efficient oxygen sensing mechanisms. The stochastic fluctuation environment favours the pOSM in all cases: the benefit of having an efficient oxygen sensing mechanism is outweighed by its cost in more randomly changing environments.

References

1 Bergmann, K., Boekelheide, N., Clarke, J. W. & et al. A billion years of temperature variability: a key driver of Earth's long-term habitability. *ESS Open Archive* (2022). https://doi.org:10.1002/essoar.10511918.3

2 Mills, B. J. W. *et al.* Modelling the long-term carbon cycle, atmospheric CO2, and Earth surface temperature from late Neoproterozoic to present day. *Gondwana Res.* **67**, 172-186 (2019). https://doi.org:https://doi.org/10.1016/j.gr.2018.12.001

3 Hancke, K. & Glud, R. N. Temperature effects on respiration and photosynthesis in three diatom-dominated benthic communities. *Aquat. Microb. Ecol.* **37**, 265-281 (2004).

4 Middelburg, J. J. *Marine Carbon Biogeochemistry*. (Springer International Publishing, 2019).

5 Li, Y.-H. & Gregory, S. Diffusion of ions in sea water and in deep-sea sediments. *Geochimica et Cosmochimica Acta* **38**, 703-714 (1974).

6 Riley, J. P. & Skirrow, G. *Chemical oceanography*. 2 edn, Vol. 1 606 (Academic Press, 1975).

7 Isaksen, M. F. & Jørgensen, B. B. Adaptation of psychrophilic and psychrotrophic sulfate-reducing bacteria to permanently cold marine environments. *Appl. Environ. Microbiol.* **62**, 408-414 (1996).

8 Raven, J. A. & Geider, R. J. Temperature and Algal Growth. *The New Phytologist* **110**, 441-461 (1988).

9 Allen, R. G., Pereira, L. S., Raes, D. & Smith, M. Crop evapotranspiration. Guidelines for computing crop water requirements. *FAO Irrigation and Drainage* **56**, 300 (1998).

10 Bartlett, B. C. & Stevenson, D. J. Analysis of a Precambrian resonance-stabilized day length. *Geophysical Research Letters* **43**, 5716-5724 (2016). https://doi.org:https://doi.org/10.1002/2016GL068912

11 García, H. E. & Gordon, L. I. Oxygen solubility in seawater: Better fitting equations. *Limnology and Oceanography* **37**, 1307-1312 (1992).

12 Langtangen, H. P. & Linge, S. *Finite Difference Computing with PDEs - A Modern Software Approach*. 507 (Springer Cham, 2017).

13 Wambecq, A. Rational Runge-Kutta methods for solving systems of ordinary differential equations. *Computing* **20**, 333-342 (1978). https://doi.org:10.1007/BF02252381

14 Norling, M. D., Jackson-Blake, L. A., Calidonio, J. L. G. & Sample, J. E. Rapid development of fast and flexible environmental models: the Mobius framework v1.0. *Geosci. Model Dev.* **14**, 1885-1897 (2021). https://doi.org:10.5194/gmd-14-1885-2021

15 Vincent, T. L. & Brown, J. S. *Evolutionary game theory, natural selection, and Darwinian dynamics*. (Cambridge University Press, 2005).

16 Hammarlund, E. U., Flashman, E., Mohlin, S. & Licausi, F. Oxygen-sensing mechanisms across eukaryotic kingdoms and their roles in complex multicellularity. *Science* **370** (2020). https://doi.org:10.1126/science.aba3512

17 Glud, R. N. Oxygen dynamics of marine sediments. *Mar. Biol. Res.* **4**, 243-289 (2008).

18 Hancke, K., Hancke, T. B., Olsen, L. M., Johnsen, G. & Glud, R. N. Temperature effects on microalgal photosynthesis-light responses measured by O2 production, pulse-amplitude-modulated fluorescence, and 14C assimilaton. *J Phycol* **44**, 501-514 (2008). https://doi.org:10.1111/j.1529-8817.2008.00487.x

19 Falkowski, P. G. & Raven, J. A. *Aquatic Photosynthesis*. (Blackwell Science, 1997).
20 Glud, R. N., Gundersen, J. K., Roy, H. & Jørgensen, B. B. Seasonal dynamics of benthic O_2 uptake in a semienclosed bay: Importance of diffusion and faunal activity. *Limnology and Oceanography* **48**, 1265-1276 (2003).
21 Middelburg, J. J., Duarte, C. M. & Gattuso, J.-P. Respiration in coastal benthic communities. *Respiration in aquatic ecosystems. Oxford University Press, Oxford*, 206-224 (2005).
22 Mayer, L. M. Surface-area control of organic-carbon accumulation in continental-shelf sediments. *Geochimica et Cosmochimica Acta* **58**, 1271-1284 (1994).
23 Sperling, E. A. & Stockey, R. G. The Temporal and Environmental Context of Early Animal Evolution: Considering All the Ingredients of an "Explosion". *Integr. Comp. Biol.* **58**, 605-622 (2018). https://doi.org:10.1093/icb/icy088
24 Davison, I. R. ENVIRONMENTAL EFFECTS ON ALGAL PHOTOSYNTHESIS: TEMPERATURE. *J. Phycol.* **27**, 2-8 (1991). https://doi.org:https://doi.org/10.1111/j.0022-3646.1991.00002.x
25 Berry, J. & Bjorkman, O. Photosynthetic response and adaptation to temperature in higher plants. *Ann. Rev. Plant Physiol.* **31**, 491-543 (1980).
26 Piiper, J. Respiratory gas exchange at lungs, gills and tissues: mechanisms and adjustments. *J Exp Biol* **100**, 5-22 (1982). https://doi.org:10.1242/jeb.100.1.5
27 Hofmann, A., Peltzer, E., Walz, P. & Brewer, P. Hypoxia by degrees: Establishing definitions for a changing ocean. *Deep Sea Res. Part I* **58**, 1212-1226 (2011).
28 Sperling, E. A., Knoll, A. H. & Girguis, P. R. The Ecological Physiology of Earth's Second Oxygen Revolution. *Annual Review of Ecology, Evolution, and Systematics* **46**, 215-235 (2015). https://doi.org:doi:10.1146/annurev-ecolsys-110512-135808
29 Canfield, D. E. & Thamdrup, B. Towards a consistent classification scheme for geochemical environments, or, why we wish the term 'suboxic' would go away. *Geobiology* **7**, 385-392 (2009). https://doi.org:10.1111/j.1472-4669.2009.00214.x
30 Scotese, C. R., Song, H., Mills, B. J. W. & van der Meer, D. G. Phanerozoic paleotemperatures: The earth's changing climate during the last 540 million years. *Earth-Sci. Rev.* **215**, 103503 (2021). https://doi.org:10.1016/j.earscirev.2021.103503
31 Vérard, C. & Veizer, J. On plate tectonics and ocean temperatures. *Geology* **47**, 881-885 (2019). https://doi.org:10.1130/g46376.1
32 Hearing, T. W. *et al.* An early Cambrian greenhouse climate. *Science Advances* **4**, eaar5690 (2018). https://doi.org:doi:10.1126/sciadv.aar5690
33 Yuan, X., Ruan, W., Bobrow, B., Carmeliet, P. & Eltzschig, H. K. Targeting hypoxia-inducible factors: therapeutic opportunities and challenges. *Nature Reviews Drug Discovery* **23**, 175-200 (2024). https://doi.org:10.1038/s41573-023-00848-6
34 Lisy, K. & Peet, D. J. Turn me on: regulating HIF transcriptional activity. *Cell Death & Differentiation* **15**, 642-649 (2008). https://doi.org:10.1038/sj.cdd.4402315
35 Huang, L. E., Gu, J., Schau, M. & Bunn, H. F. Regulation of hypoxia-inducible factor 1alpha is mediated by an O2-dependent degradation domain via the ubiquitin-proteasome pathway. *Proc Natl Acad Sci U S A* **95**, 7987-7992 (1998). https://doi.org:10.1073/pnas.95.14.7987
36 Maxwell, P. H. *et al.* The tumour suppressor protein VHL targets hypoxia-inducible factors for oxygen-dependent proteolysis. *Nature* **399**, 271-275 (1999). https://doi.org:10.1038/20459

37 Iwai, K. *et al.* Identification of the von Hippel-lindau tumor-suppressor protein as part of an active E3 ubiquitin ligase complex. *Proc Natl Acad Sci U S A* **96**, 12436-12441 (1999). https://doi.org:10.1073/pnas.96.22.12436

38 Lando, D. *et al.* FIH-1 is an asparaginyl hydroxylase enzyme that regulates the transcriptional activity of hypoxia-inducible factor. *Genes Dev.* **16**, 1466-1471 (2002). https://doi.org:10.1101/gad.991402

39 Graham, A. M. & Presnell, J. S. Hypoxia Inducible Factor (HIF) transcription factor family expansion, diversification, divergence and selection in eukaryotes. *PloS one* **12**, e0179545 (2017).

40 Mills, D. B. *et al.* The last common ancestor of animals lacked the HIF pathway and respired in low-oxygen environments. *eLife* **7**, e31176 (2018). https://doi.org:10.7554/eLife.31176
