# Supplementary material for: Benthic diel oxygen variability and stress as potential drivers for animal diversification in the Neoproterozoic-Palaeozoic
Source: Nat Commun. 2025 Mar 21;16:2223. doi: 10.1038/s41467-025-57345-0 (PMC11928486; doi:10.1038/s41467-025-57345-0)
Supplement: Supplementary file 2 — Description of Additional Supplementary Files [file 41467_2025_57345_MOESM2_ESM.pdf]

Description of Additional Supplementary Files

Supplementary Code 1

Code for biogeochemical model (Mobius framework).

Supplementary Code 2

Code for model of population dynamics and speed of phenotypic plasticity
Code for competition experiment
