# Supplementary material for: Benthic diel oxygen variability and stress as potential drivers for animal diversification in the Neoproterozoic-Palaeozoic
Source: Nat Commun. 2025 Mar 21;16:2223. doi: 10.1038/s41467-025-57345-0 (PMC11928486; doi:10.1038/s41467-025-57345-0)
Supplement: Supplementary file 3 — Supplementary Code 1-2 [file 41467_2025_57345_MOESM3_ESM.zip › Supplementary Code file 1.docx]

inline double

Diffusion(double Z, double Zlow, double Zupp, double Dlow, double Dupp, double C, double Clow, double Cupp)

{

//Computes the term of dC(Z,t)/dt that comes from diffusion.

double Zupphalf = 0.5*(Z + Zupp);

double Zlowhalf = 0.5*(Z + Zlow);

double Flow = Dlow * (Clow - C) / (Zlow - Z);

double Fupp = Dupp * (Cupp - C) / (Zupp - Z);

return (Fupp - Flow) / (Zupphalf - Zlowhalf);

}

inline double

DynamicViscosity(double Temperature, double Salinity)

{

#if 1

// Formula from J.P. Riley, Chemical Oceanography, vol 4, 2nd edition. AP. 1975

//Temperature: deg C, Salinity: PSU (PSS-78)

// return unit Pa*s

//NOTE: It is also possible to add pressure corrections. Pressure is here assumed to be at 1atm.

double eta20 = 1.0020; //viscosity at t=20, s=0

double t = Temperature;

double tm20 = 20.0-t;

double logratio = (1.1709*tm20 - 0.001827*tm20*tm20) / (t + 89.93);

// Dynamic viscosity of distilled water corrected for temperature

double eta_t = eta20*pow(10.0, logratio);

double A = LinearInterpolate(t, 5.0, 25.0, 0.000366, 0.001403);

double B = LinearInterpolate(t, 5.0, 25.0, 0.002756, 0.003416);

double dtemp = (t + 273.15 - 277.13); // Difference between temperature and reference temperature

double density = 999.98*(1.0 - 0.5*1.6509e-5*dtemp*dtemp); // density of water (kg/m3)

double Cl = Max((Salinity - 0.03)/1.805, 0.0); // The given formula is actually Salinity = 0.03 + 1.805*Cl, but that does not allow for 0 salinity.

// Volume chlorinity:

double Clv = density*1e-3 * Cl; //Density should apparently be in kg/l instead of kg/m3 for this formula even though it is not stated.

// Dynamic viscosity corrected for salinity

double eta_t_s = eta_t*(1.0 + A*sqrt(Clv) + B*Clv);

return eta_t_s * 1e-3; //Convert centipoise -> Pa*s

#else

return 2.414e-5*pow(10.0, 247.8 / (Temperature+273.15 - 140.0));

#endif

}

inline double

Respiration(double Rate, double Conc, double Threshold)

{

if(Conc < Threshold)

{

return Rate * (Conc / Threshold);

}

return Rate;

}

void

AddSedimentOxygenModule(mobius_model *Model)

{

BeginModule(Model, "Sediment oxygen", "0.0");

auto Dimensionless = RegisterUnit(Model);

auto PSS = RegisterUnit(Model, "PSS-78");

auto DegreesCelsius = RegisterUnit(Model, "¬∞C");

auto UMolsPerM2PerS = RegisterUnit(Model, "¬µmol(photons)/m2/s");

auto PercentAtm = RegisterUnit(Model, "% atm");

auto Cm2PerS = RegisterUnit(Model, "cm2/s");

auto Cm = RegisterUnit(Model, "cm");

auto M = RegisterUnit(Model, "m");

auto PerM = RegisterUnit(Model, "1/m");

auto Degrees = RegisterUnit(Model, "¬∞");

auto WPerM2 = RegisterUnit(Model, "W/m2");

auto UMolsPerCm2PerS = RegisterUnit(Model, "¬µmol(oxygen)/cm2/s");

auto UMolsPerCm3PerS = RegisterUnit(Model, "¬µmol(oxygen)/cm3/s");

auto UMolsPerUMols = RegisterUnit(Model, "¬µmol(oxygen)/¬µmol(photons)");

auto UMolsPerUMolsPerS = RegisterUnit(Model, "¬µmol(oxygen)/¬µmol(TOC)/s");

auto UMolsPerCm3 = RegisterUnit(Model, "¬µmol(oxygen)/cm3");

auto UMolsTOCPerCm3 = RegisterUnit(Model, "¬µmol(TOC)/cm3");

auto PerS = RegisterUnit(Model, "1/s");

auto Days = RegisterUnit(Model, "days");

auto Hours = RegisterUnit(Model, "hours");

auto SedimentOx = RegisterParameterGroup(Model, "Sediment oxygen");

//TODO: Find good default values:

auto AtmosphericO2Saturation = RegisterParameterDouble(Model, SedimentOx, "Atmospheric O2 partial pressure", PercentAtm, 21.0, 0.0, 100.0);

auto Temperature = RegisterParameterDouble(Model, SedimentOx, "Temperature", DegreesCelsius, 20.0, -5.0, 40.0);

auto Salinity = RegisterParameterDouble(Model, SedimentOx, "Salinity", PSS, 35.0, 0.0, 1000.0);

auto Latitude = RegisterParameterDouble(Model, SedimentOx, "Latitude", Degrees, 60.0, -90.0, 90.0);

auto YearLength = RegisterParameterDouble(Model, SedimentOx, "Year length", Days, 356.0, 200.0, 500.0);

auto DayLength = RegisterParameterDouble(Model, SedimentOx, "Day length", Hours, 24.0, 15.0, 30.0);

auto CloudCover = RegisterParameterDouble(Model, SedimentOx, "Average cloud cover", Dimensionless, 0.0, 0.0, 1.0);

auto SedimentPorosity = RegisterParameterDouble(Model, SedimentOx, "Sediment porosity", Dimensionless, 0.5, 0.0, 1.0);

auto AttenuationCoefficient = RegisterParameterDouble(Model, SedimentOx, "Attenuation coefficient", PerM, 0.01, 0.0, 0.2);

auto LuminosityConversionFactor = RegisterParameterDouble(Model, SedimentOx, "Luminosity conversion factor", Dimensionless, 4.1, 0.0, 5.0, "¬µmol(photons)/s per W (depends on desired PAR spectrum)"); //TODO: Unit

auto MaxProduction = RegisterParameterDouble(Model, SedimentOx, "Maximum O2 production at 20¬∞C", UMolsPerCm2PerS, 0.0, 0.0, 1000.0);

auto MaxLightUtilization = RegisterParameterDouble(Model, SedimentOx, "Maximum light utilization", UMolsPerUMols, 0.0, 0.0, 10.0);

auto Q10Prod = RegisterParameterDouble(Model, SedimentOx, "Production rate response to a 10¬∞ change in temperature (Q10)", Dimensionless, 2.0, 1.0, 4.0);

auto RespirationAt20Degrees = RegisterParameterDouble(Model, SedimentOx, "Respiration rate at 20¬∞C", UMolsPerUMolsPerS, 0.0, 0.0, 0.01);

auto TOCConcentration = RegisterParameterDouble(Model, SedimentOx, "TOC concentration", UMolsTOCPerCm3, 0.0, 0.0, 0.1);

auto Q10Resp = RegisterParameterDouble(Model, SedimentOx, "Respiration rate response to a 10¬∞ change in temperature (Q10)", Dimensionless, 2.0, 1.0, 4.0);

auto RespirationCutoffThreshold = RegisterParameterDouble(Model, SedimentOx, "Respiration cutoff threshold", UMolsPerCm3, 10.0, 0.01, 100.0);

auto OceanDepth = RegisterParameterDouble(Model, SedimentOx, "Ocean depth", M, 10.0, 0.0, 200.0);

//auto DBLThickness = RegisterParameterDouble(Model, SedimentOx, "Thickness of diffusive boundary layer", Cm, 5.0, 0.0, 30.0);

//auto ProductionZoneThickness = RegisterParameterDouble(Model, SedimentOx, "Thickness of production zone", Cm, 5.0, 0.0, 30.0);

//auto NonProductionZoneThickness = RegisterParameterDouble(Model, SedimentOx, "Thickness of non-production zone", Cm, 50.0, 0.0, 200.0);

auto UpperProductionIndex = RegisterParameterUInt(Model, SedimentOx, "Upper layer of production zone", Dimensionless, 5, 1, 100, "Which of the layers are the topmost one belonging to production zone");

auto LowerProductionIndex = RegisterParameterUInt(Model, SedimentOx, "Lower layer of production zone", Dimensionless, 6, 1, 100, "Which of the layers are the lowest one belonging to the production zone");

auto ZResolution = RegisterParameterDouble(Model, SedimentOx, "Z resolution", Cm, 0.1, 0.001, 1.0, "Thickness of each computed layer");

auto SurfaceSolarRadiation = RegisterEquation(Model, "Surface solar radiation", WPerM2);

auto BottomSolarRadiation = RegisterEquation(Model, "Bottom solar radiation", WPerM2);

auto Luminosity = RegisterEquation(Model, "Bottom luminosity", UMolsPerM2PerS);

auto OxygenProductionRate = RegisterEquation(Model, "Oxygen production rate", UMolsPerCm3PerS);

auto OxygenRespirationRate = RegisterEquation(Model, "Oxygen respiration rate", PerS);

auto OxygenDiffusivityInWater = RegisterEquation(Model, "Oxygen diffusivity in water", Cm2PerS);

EQUATION(Model, SurfaceSolarRadiation,

//Based on standard theory for extraterrestrial radiation, but modified for different length of day and length of year.

s64 Seconds = CURRENT_TIME().DateTime.SecondsSinceEpoch;

double DayLen = PARAMETER(DayLength)*3600.0; //seconds

double Day = (double)Seconds / DayLen;

double YearLen = PARAMETER(YearLength); //days

double Dum;

double DayAngle = 2.0*Pi*modf(Day / YearLen, &Dum);

double SecondOfDay = modf(Day, &Dum)*DayLen;

//TODO: This is probably not correct at all for the given time period....

double DeclinationAngle = 0.409*sin(DayAngle - 1.39);

double LocalTime = SecondOfDay / 3600.0;

double SolarTime1 = LocalTime;// + ET/60.0;// + (4.0/60.0)*(PARAMETER(StandardMeridianLocalZone) - PARAMETER(Longitude)); //NOTE: Simplify

double SolarTime2 = SolarTime1 + 1.0;

double SolarHourAngle1 = 15.0*(SolarTime1 - 12.0)*Pi/180.0;

double SolarHourAngle2 = 15.0*(SolarTime2 - 12.0)*Pi/180.0;

double SolarConstant = 1367.0; // W/m2

//TODO: Is probably also incorrect...

double InverseRelativeDistanceEarthSun = 1.0 + 0.033*cos(DayAngle);

double lat = PARAMETER(Latitude)*Pi/180.0;

double SRad = (12.0 * SolarConstant * InverseRelativeDistanceEarthSun / Pi)*

(

sin(lat)*sin(DeclinationAngle)*(SolarHourAngle2 - SolarHourAngle1)

+ cos(lat)*cos(DeclinationAngle)*(sin(SolarHourAngle2) - sin(SolarHourAngle1))

);

SRad = Max(SRad, 0.0);

return SRad * 0.75 * (1.0 - 0.65*PARAMETER(CloudCover)); //Note very simple correction for atmosphere and cloud cover

)

EQUATION(Model, BottomSolarRadiation,

return RESULT(SurfaceSolarRadiation)*exp(-PARAMETER(AttenuationCoefficient)*PARAMETER(OceanDepth));

)

EQUATION(Model, Luminosity,

return RESULT(BottomSolarRadiation)*PARAMETER(LuminosityConversionFactor);

)

EQUATION(Model, OxygenProductionRate,

//In production zone only

double production_zone_thickness = ((double)PARAMETER(LowerProductionIndex) - (double)PARAMETER(UpperProductionIndex) + 1.0)*PARAMETER(ZResolution);

double temprate = pow(PARAMETER(Q10Prod), (PARAMETER(Temperature) - 20.0)/10.0);

return temprate * PARAMETER(MaxProduction)*(1.0 - exp(-PARAMETER(MaxLightUtilization)*1e4*RESULT(Luminosity)/PARAMETER(MaxProduction))) / production_zone_thickness;

)

EQUATION(Model, OxygenRespirationRate,

return PARAMETER(RespirationAt20Degrees)*PARAMETER(TOCConcentration)*pow(PARAMETER(Q10Resp), (PARAMETER(Temperature) - 20.0)/10.0);

)

EQUATION(Model, OxygenDiffusivityInWater,

double RefDiff = 1.57e-5; //Reference diffusivity at 10C, distilled water

//TODO: Formula of temperature and salinity

double t = PARAMETER(Temperature);

double s = PARAMETER(Salinity);

return RefDiff * (DynamicViscosity(10.0, 0.0) / DynamicViscosity(t, s)) * (t+273.15)/283.15;

)

auto SedSolver = RegisterSolver(Model, "Sediment solver", 0.1, IncaDascru);

auto Conc0 = RegisterEquation(Model, "C0", UMolsPerCm3);

EQUATION(Model, Conc0,

//NOTE: Garcia & Gordon 92

double A0 = 5.80818;

double A1 = 3.20684;

double A2 = 4.11890;

double A3 = 4.93845;

double A4 = 1.01567;

double A5 = 1.41575;

double B0 = -7.01211e-03;

double B1 = -7.25958e-03;

double B2 = -7.93334e-03;

double B3 = -5.54491e-03;

double C = -1.32412e-07;

double Ts = log((298.15 - PARAMETER(Temperature)) / (273.15 + PARAMETER(Temperature)));

double AA = ((((A5*Ts + A4)*Ts + A3)*Ts + A2)*Ts + A1)*Ts + A0;

double BB = ((B3*Ts + B2)*Ts + B1)* Ts + B0;

double S = PARAMETER(Salinity);

double O2 = exp(AA + S*(BB + S*C));

//Correct for different atmospheric partial pressure

O2 = O2 * PARAMETER(AtmosphericO2Saturation) / 21.0;

return O2*1025.0*1e-6; //Convert 1/kg to 1/cm3. TODO: Ooops, does not account for variations in water density!

)

#define LevelEq(Dum1, LevIDX, Dum2) \

auto Conc##LevIDX = RegisterEquationODE(Model, "C"#LevIDX , UMolsPerCm3, SedSolver);

#include "Levels.h"

auto Conc101 = RegisterEquation(Model, "C101", UMolsPerCm3);

EQUATION(Model, Conc101,

return 0.0;

)

#undef LevelEq

#define LevelEq(LevIDXLow, LevIDX, LevIDXUp) \

EQUATION(Model, Conc##LevIDX, \

double Zres = PARAMETER(ZResolution); \

double Z = (double)LevIDX*Zres; \

double Zupp = Z + Zres; \

double Zlow = Z - Zres; \

double Cupp = RESULT(Conc##LevIDXUp); \

double C = RESULT(Conc##LevIDX); \

double Clow = RESULT(Conc##LevIDXLow); \

double D0 = RESULT(OxygenDiffusivityInWater); \

double D = D0*PARAMETER(SedimentPorosity); \

double Dupp = LevIDX <= PARAMETER(UpperProductionIndex) ? D0 : D; \

double Dlow = LevIDX < PARAMETER(UpperProductionIndex) ? D0 : D; \

double diff = Diffusion(Z, Zlow, Zupp, Dlow, Dupp, C, Clow, Cupp); \

double prod = RESULT(OxygenProductionRate); \

if(LevIDX < PARAMETER(UpperProductionIndex) || LevIDX > PARAMETER(LowerProductionIndex)) prod = 0.0; \

double resp = Respiration(RESULT(OxygenRespirationRate), C, PARAMETER(RespirationCutoffThreshold)); \

return (diff + prod - resp)*3600.0; \

)

#include "Levels.h"

#undef LevelEq

EndModule(Model);

}
