# Supplementary material for: Benthic diel oxygen variability and stress as potential drivers for animal diversification in the Neoproterozoic-Palaeozoic
Source: Nat Commun. 2025 Mar 21;16:2223. doi: 10.1038/s41467-025-57345-0 (PMC11928486; doi:10.1038/s41467-025-57345-0)
Supplement: Supplementary file 3 — Supplementary Code 1-2 [file 41467_2025_57345_MOESM3_ESM.zip › Supplementary Code file 2.docx]

**Supplementary Code**

1. **Code for model of population dynamics and speed of phenotypic plasticity**
2. **Code for competition experiment**
3. Code for model of population dynamics and speed of phenotypic plasticity:

#!/usr/bin/env python

# coding: utf-8

# In[17]:

import numpy as np

from scipy.integrate import *

import matplotlib.pyplot as plt

import math

from math import exp

pop1 = 10

pop2 = 10

strat1 = strat2 = 0

time = 2000

KM = 100

d = 0.04

r = 0.25

k1 = 0.2

k2 = .5

g = np.random.random_sample(time+1)*4-2

h = np.random.random_sample(time+1)*10-5

g1 = []

h1 = []

for i in range(len(g)):

if (i%10==0):

g1.append(g[i])

h1.append(h[i])

else:

g1.append(g[i-1])

h1.append(h[i-1])

IC = [pop1,pop2,strat1,strat2]

sk = 12.5

def evoLV(X, t):

if t<500:

gamma = np.sin(t/50) + g1[int(t)]

else:

gamma = np.sin(t/50) + h1[int(t)] #+ 5*np.sin(t/50) #h1 instead of g1 for stochastic, sin term for periodic

x1 = X[0]

x2 = X[1]

u1 = X[2]

u2 = X[3]

if x1<.1:

x1=0

if x2<.1:

x2=0

K1 = KM * math.exp(-((u1 - gamma) ** 2) / (2 * sk))

K2 = KM * math.exp(-((u2 - gamma) ** 2) / (2 * sk))

dx1dt = x1 * (r/K1 * (K1 - x1 - x2) - d*k1)

dx2dt = x2 * (r/K2 * (K2 - x1 - x2) - d*k2)

dG1dv = -r*(-2*gamma + 2*u1)/(2*sk) + r*(-2*gamma + 2*u1)*(KM*exp(-(-gamma + u1)**2/(2*sk)) - x1 - x2)*exp((-gamma + u1)**2/(2*sk))/(2*KM*sk)

dG2dv = -r*(-2*gamma + 2*u2)/(2*sk) + r*(-2*gamma + 2*u2)*(KM*exp(-(-gamma + u2)**2/(2*sk)) - x1 - x2)*exp((-gamma + u2)**2/(2*sk))/(2*KM*sk)

dv1dt = k1 * dG1dv

dv2dt = k2 * dG2dv

dxvdt = np.array([dx1dt, dx2dt, dv1dt, dv2dt])

return dxvdt

intxv = np.array(IC)

pop = odeint(evoLV, intxv, range(time+1))

plt.figure()

plt.subplot(211)

plt.title('Stochastic & Periodic Fluctuations')

plt.plot(pop[:,0],'dimgrey',lw=3)

plt.plot(pop[:,1],'goldenrod',lw=3)

plt.ylabel('Pop Size, x')

ax = plt.gca()

plt.subplot(212)

plt.plot(pop[:,2],'dimgrey',lw=3)

plt.plot(pop[:,3],'goldenrod',lw=3)

plt.ylabel('Indv Strategy, v')

ax = plt.gca()

plt.show()

# In[ ]:

1. Code for competition experiment:

import numpy as np

import matplotlib.pyplot as plt

from matplotlib.lines import Line2D

# Define the data sets

dlev = [0, 0.01, 0.02, 0.03, 0.04, 0.05, 0.06, 0.07, 0.08, 0.09, 0.1]

stochastic = [-3311, 5628, 1448, 860, 615, 484, 402, 337, 301, 263, 230]

periodic = [-582, -691, -710, -851, -906, -1060, -1488, -1990, -3426, 262, 230]

stoch_period = [-573, -609, -676, -698, -762, -843, -991, -1035, -1204, -1647, -2106]

# Define colors for positive and negative values

positive_color = 'b'  # Blue for positive values

negative_color = 'r'  # Red for negative values

# Create subplots

plt.figure(figsize=(10, 6))

# Set marker size

marker_size = 100

# Create scatter plot for stochastic with absolute values

stochastic_plot = plt.scatter(dlev, np.abs(stochastic), c=np.where(np.array(stochastic) >= 0, positive_color, negative_color), marker='o', s=marker_size, label='Stochastic Fluctuations')

# Create scatter plot for periodic with absolute values

periodic_plot = plt.scatter(dlev, np.abs(periodic), c=np.where(np.array(periodic) >= 0, positive_color, negative_color), marker='x', s=marker_size, label='Periodic Fluctuations')

# Create scatter plot for stoch_period with absolute values

stoch_period_plot = plt.scatter(dlev, np.abs(stoch_period), c=np.where(np.array(stoch_period) >= 0, positive_color, negative_color), marker='^', s=marker_size, label='Stochastic and Periodic Fluctuations')

# Create custom legend handles with black colors

legend_elements = [

    Line2D([0], [0], marker='o', color='k', label='Stochastic Fluctuations', markersize=10),

    Line2D([0], [0], marker='x', color='k', label='Periodic Fluctuations', markersize=10),

    Line2D([0], [0], marker='^', color='k', label='Stochastic and Periodic Fluctuations', markersize=10),

]

# Create a legend for positive values in blue

positive_legend = Line2D([0], [0], marker='s', color='b', label='eOSM Extinction', markersize=10)

negative_legend = Line2D([0], [0], marker='s', color='r', label='pOSM Extinction', markersize=10)

# Add the custom legend to the plot

plt.legend(handles=legend_elements + [positive_legend, negative_legend])

# Set axis labels and a title

plt.xlabel('Cost of oxygen sensing mechanism, $d$')

plt.ylabel('Extinction Time')

plt.title('Extinction Times for eOSM and pOSM Species under Different Environmental Conditions')

# Show the plot

plt.show()
