# Supplementary material for: Benthic diel oxygen variability and stress as potential drivers for animal diversification in the Neoproterozoic-Palaeozoic
Source: Nat Commun. 2025 Mar 21;16:2223. doi: 10.1038/s41467-025-57345-0 (PMC11928486; doi:10.1038/s41467-025-57345-0)
Supplement: Supplementary file 4 — Reporting Summary [file 41467_2025_57345_MOESM4_ESM.pdf]

nature portfolio

Corresponding author(s): Hammarlund, E.U., NCOMMS-24-28343A

Last updated by author(s): Feb 12, 2025

Reporting Summary

Nature Portfolio wishes to improve the reproducibility of the work that we publish. This form provides structure for consistency and transparency in reporting. For further information on Nature Portfolio policies, see our Editorial Policies and the Editorial Policy Checklist.

Statistics

For all statistical analyses, confirm that the following items are present in the figure legend, table legend, main text, or Methods section.

n/a	Confirmed	
☐	☒	The exact sample size (*n*) for each experimental group/condition, given as a discrete number and unit of measurement
☒	☐	A statement on whether measurements were taken from distinct samples or whether the same sample was measured repeatedly
☒	☐	The statistical test(s) used AND whether they are one- or two-sided<br>*Only common tests should be described solely by name; describe more complex techniques in the Methods section.*
☐	☒	A description of all covariates tested
☒	☐	A description of any assumptions or corrections, such as tests of normality and adjustment for multiple comparisons
☒	☐	A full description of the statistical parameters including central tendency (e.g. means) or other basic estimates (e.g. regression coefficient) AND variation (e.g. standard deviation) or associated estimates of uncertainty (e.g. confidence intervals)
☒	☐	For null hypothesis testing, the test statistic (e.g. *F*, *t*, *r*) with confidence intervals, effect sizes, degrees of freedom and *P* value noted<br>*Give P values as exact values whenever suitable.*
☐	☒	For Bayesian analysis, information on the choice of priors and Markov chain Monte Carlo settings
☐	☒	For hierarchical and complex designs, identification of the appropriate level for tests and full reporting of outcomes
☐	☒	Estimates of effect sizes (e.g. Cohen's *d*, Pearson's *r*), indicating how they were calculated

Our web collection on statistics for biologists contains articles on many of the points above.

Software and code

Policy information about availability of computer code

Data collection	Data collection pertains to literature and database studies to chart the individual ranges of those parameters (e.g., TOC, temperature, diffusion, shelf area) that are relevant for the biogeochemical model. Some of these ranges are constrained for modern conditions and, later, inferred for their likely ranges in the Paleozoic.
Data analysis	To explore daily oxygen dynamics at the sediment–water interface at varying temperatures and carbon loads, we solve a 1D partial differential equation implemented in the Mobius framework. We also use the G functions framework to model the ecological (population) and evolutionary (strategy) dynamics of species with effective and poor oxygen-sensing mechanisms existing in the same environment.

For manuscripts utilizing custom algorithms or software that are central to the research but not yet described in published literature, software must be made available to editors and reviewers. We strongly encourage code deposition in a community repository (e.g. GitHub). See the Nature Portfolio guidelines for submitting code & software for further information.

Data

Policy information about availability of data

All manuscripts must include a data availability statement. This statement should provide the following information, where applicable:

- Accession codes, unique identifiers, or web links for publicly available datasets
- A description of any restrictions on data availability
- For clinical datasets or third party data, please ensure that the statement adheres to our policy

Supplementary information is provided. The model is implemented using the Mobius framework, and the source code can be found at [github]. Files used for

running the model can be found at [github].

Research involving human participants, their data, or biological material

Policy information about studies with human participants or human data. See also policy information about sex, gender (identity/presentation), and sexual orientation and race, ethnicity and racism.

Reporting on sex and gender	n/a
Reporting on race, ethnicity, or other socially relevant groupings	n/a
Population characteristics	n/a
Recruitment	n/a
Ethics oversight	n/a

Note that full information on the approval of the study protocol must also be provided in the manuscript.

Field-specific reporting

Please select the one below that is the best fit for your research. If you are not sure, read the appropriate sections before making your selection.

☐ Life sciences ☐ Behavioural & social sciences ☒ Ecological, evolutionary & environmental sciences

For a reference copy of the document with all sections, see nature.com/documents/nr-reporting-summary-flat.pdf

Ecological, evolutionary & environmental sciences study design

All studies must disclose on these points even when the disclosure is negative.

Study description	In this study, we used a biogeochemical model to consider oxygen dynamics at the sunlit sediment–water interface over night-day cycles in the early Cambrian under a greenhouse climate.
Research sample	The research samples are modeling results from a) a biogeochemical model and b) a mathematical G-function model. This is chosen since it allows us to constrain the daily oxygen dynamics over a large parameter space (run simulations as parameters change) and the cost-benefit trade-offs that are associated with coping with oxygen dynamics of different amplitudes.
Sampling strategy	N/A
Data collection	Data collection from the biogeochemical model occurs online when using the Mobius framework. It was performed and analyzed by EUH, MN, and KH. Data collection from the G-function framework was performed locally by AB.
Timing and spatial scale	The models were continuously developed over 2019-2023.
Data exclusions	No data was excluded.
Reproducibility	All modeling results are reproducible and the Monte-Carlo simulation of the biogeochemical model explores a wide parameter space. This simulation offers an estimation of the extent when our presumed conditions are fulfilled.
Randomization	Randomization was not applicable in these modeling experiments.
Blinding	Blinding was not possible since the study is a modeling exercise. However, the Monte-Carlo simulation of the biogeochemical results takes into account the ranges of individual parameters, and in that sense offers an estimation of the extent when our presumed conditions are fulfilled.

Did the study involve field work? ☐ Yes ☒ No

Reporting for specific materials, systems and methods

We require information from authors about some types of materials, experimental systems and methods used in many studies. Here, indicate whether each material, system or method listed is relevant to your study. If you are not sure if a list item applies to your research, read the appropriate section before selecting a response.

Materials & experimental systems

n/a	Involved in the study
☒	☐ Antibodies
☒	☐ Eukaryotic cell lines
☒	☐ Palaeontology and archaeology
☒	☐ Animals and other organisms
☒	☐ Clinical data
☒	☐ Dual use research of concern
☒	☐ Plants

Methods

n/a	Involved in the study
☒	☐ ChIP-seq
☒	☐ Flow cytometry
☒	☐ MRI-based neuroimaging
